# Doubling survival and improving clinical outcomes using a left ventricular assist device instead of chest compressions for resuscitation after prolonged cardiac arrest: a large animal study

**DOI:** 10.1186/s13054-015-0864-2

**Published:** 2015-03-26

**Authors:** Matthias Derwall, Anne Brücken, Christian Bleilevens, Andreas Ebeling, Philipp Föhr, Rolf Rossaint, Karl B Kern, Christoph Nix, Michael Fries

**Affiliations:** Klinik für Anästhesiologie, Uniklinik RWTH Aachen, Pauwelsstrasse 30, Aachen, D-52074 Germany; Division of Cardiology, University of Arizona College of Medicine, 1501 North Campbell Avenue, Tucson, AZ 85724 USA; Abiomed Europe GmbH, Neuenhofer Weg 3, Aachen, D-52074 Germany

## Abstract

**Introduction:**

Despite improvements in pre-hospital and post-arrest critical care, sudden cardiac arrest (CA) remains one of the leading causes of death. Improving circulation during cardiopulmonary resuscitation (CPR) may improve survival rates and long-term clinical outcomes after CA.

**Methods:**

In a porcine model, we compared standard CPR (sCPR; n =10) with CPR using an intravascular cardiac assist device without additional chest compressions (iCPR; n =10) following 10 minutes of electrically induced ventricular fibrillation (VF). In a separate crossover experiment, 10 additional pigs were subjected to 10 minutes of VF and 6 minutes of sCPR; the iCPR device was then implanted if a return of spontaneous circulation (ROSC) was not achieved using sCPR. Animals were evaluated in respect to intra- and post-arrest hemodynamics, survival, functional outcome and cerebral and myocardial lesions following CPR. We hypothesized that iCPR would result in more frequent ROSC and better functional recovery than sCPR.

**Results:**

iCPR produced a mean flow of 1.36 ± 0.02 L/min, leading to significantly higher coronary perfusion pressure (CPP) values during the early period of CPR (22 ± 10 mmHg vs. 9 ± 5 mmHg, *P* ≤0.01, 1 minute after start of CPR; 20 ± 11 mmHg vs. 10 ± 7 mmHg, *P* =0.03, 2 minutes after start of CPR), resulting in high ROSC rates (100% in iCPR vs. 50% in sCPR animals; *P* =0.03). iCPR animals showed significantly lower serum S100 levels at 10 and 30 minutes following ROSC (3.5 ± 0.6 ng/ml vs. 7.4 ± 3.0 ng/ml 30 minutes after ROSC; *P* ≤0.01), as well as superior clinical outcomes based on overall performance categories (2.9 ± 1.0 vs. 4.6 ± 0.8 on day 1; *P* ≤0.01). In crossover experiments, 80% of animals required treatment with iCPR after failed sCPR. Notably, ROSC was still achieved in six of the remaining eight animals (75%) after a total of 22.8 ± 5.1 minutes of ischemia.

**Conclusions:**

In a model of prolonged cardiac arrest, the use of iCPR instead of sCPR improved CPP and doubled ROSC rates, translating into improved clinical outcomes.

## Introduction

Chest compression, assisted ventilation and defibrillation provide the basis of modern cardiopulmonary resuscitation (CPR). However, a large proportion of patients with cardiac arrest die despite resuscitation efforts or survive with detrimental functional outcomes [1,2]. Prolonged ischemia and the limited efficacy of conventional CPR measures have been identified as contributors to the high cardiac arrest mortality, which exceeds 70% [3,4]; thus international guidelines are aimed at promoting the importance of uninterrupted, high-quality chest compressions [5]. Unfortunately, manual chest compressions result in only 25% to 40% of pre-arrest cardiac output values [6], and compressions are often interrupted [7]. Thus, improving circulation during CPR may increase the number of successfully resuscitated patients. However, recent studies of the use of external mechanical resuscitation devices have not demonstrated consistent improvement in outcomes [8-10].

**Figure 1:**
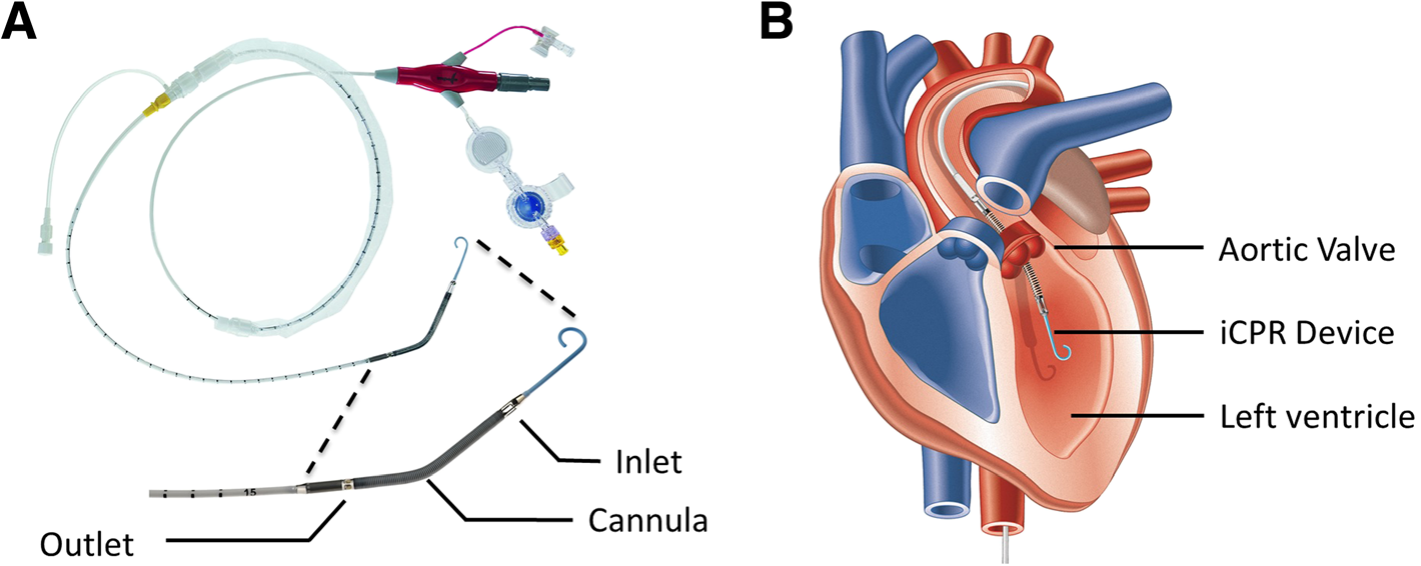
**Photograph of the Impella 2.5 intravascular cardiopulmonary resuscitation device and illustration of its intraventricular placement. (A)** Photograph of the unmodified percutaneous left ventricular assist device (Impella 2.5; Abiomed Europe GmbH, Aachen, Germany) used in this investigation. The lower portion of the picture is a magnification of the catheter tip. **(B)** Illustration of the final position of the device with the tip in the left ventricle. Note that the inlet area of the pump is positioned within the left ventricle, with the outlet situated in the ascending aorta, serving both the coronary and carotid arteries. Both pictures are reprinted with permission from Abiomed. iCPR, Intravascular cardiopulmonary resuscitation.

Mechanical circulatory support is increasingly being used in patients with acute heart failure [11]. Recently, the use of venoarterial extracorporeal membrane oxygenation (ECMO) has been reproposed [12-14] to improve survival in patients presenting with in-hospital cardiac arrest [14]. However, the emergency use of ECMO requires insertion of large-bore cannulas into the femoral artery and vein and priming a complex system including tubing and a centrifugal pump. In contrast, the use of a minimally invasive intravascular left ventricular assist device (Impella 2.5; Abiomed, Danvers, MA, USA), which pumps blood from the left ventricle into the ascending aorta, requires only a single vascular access with no external tubing (Figure 1). The device is used to support patients during high-risk percutaneous interventions, post-cardiotomy heart failure and severe cardiogenic shock [15]. In previous experimental studies, researchers demonstrated superior perfusion of vital organs in a cardiac arrest setting in an open-chest pig model using the Impella 2.5 compared with direct cardiac compression [16]. Whether such a strategy could be superior to standard CPR (sCPR) in a closed-chest model with respect to improved CPR success rates and better neurological outcomes is unknown. Therefore, we designed this study to test the efficacy of intravascular CPR (iCPR) using the Impella 2.5 in an established porcine model of cardiac arrest. We hypothesized that iCPR would result in a more frequent return of spontaneous circulation (ROSC) and better functional recovery compared with sCPR.

## Material and methods

### Study design and ethical approval

A large animal model of cardiac arrest and CPR was employed, as has been previously described by our group [17-20]. Thirty male domestic pigs at 4 months of age, (weighing 34.6 ± 4.6 kg) were studied. All procedures were conducted in accordance with principles set forth for the care and use of animals based on the Helsinki Declaration, and they were approved by the appropriate governmental institution (Landesamt für Natur, Umwelt und Verbraucherschutz NRW (LANUV), Recklinghausen, Germany).

### Anesthesia and instrumentation

Pigs were anesthetized with an intramuscular injection of 4 mg/kg azaperone, followed by ear vein injection of 15 mg/kg sodium pentobarbital. Anesthetized animals were intubated and mechanically ventilated (Servo Ventilator 300A; Siemens AG, Munich, Germany) with an inspired oxygen fraction (FiO_2_) of 0.21 and a tidal volume of 10 ml/kg. The respiratory frequency was adjusted to maintain an end-tidal carbon dioxide tension (pCO_2_) between 35 and 40 mmHg (4.7 and 5.3 kPa). A continuous infusion of pentobarbital (4 mg/kg/hr) was administered during the preparation period and discontinued 30 minutes before the induction of ventricular fibrillation (VF). Anesthesia was resumed 30 minutes after successful resuscitation and continued until animals were weaned from the respirator. Placement of indwelling catheters in femoral artery and vein was performed with ultrasound guidance. Finally, a HexaLumen Swan-Ganz catheter was flow-directed from the left femoral vein into the pulmonary artery (744HF75; Edwards Lifesciences, Irvine, CA, USA). To induce VF, a 5-French pacing catheter was fluoroscopically advanced from the surgically exposed left cephalic vein into the right ventricle. Blood temperature (measured in the pulmonary artery via the Swan-Ganz catheter) was maintained at 38.2 ± 0.2°C during preparation using a convective heating blanket (Warm Touch 5200; Tyco Healthcare, Pleasanton, CA, USA). To ensure adequate hydration, a continuous infusion of Ringer’s solution was administered at 4 ml/kg/hr.

**Figure 2:**
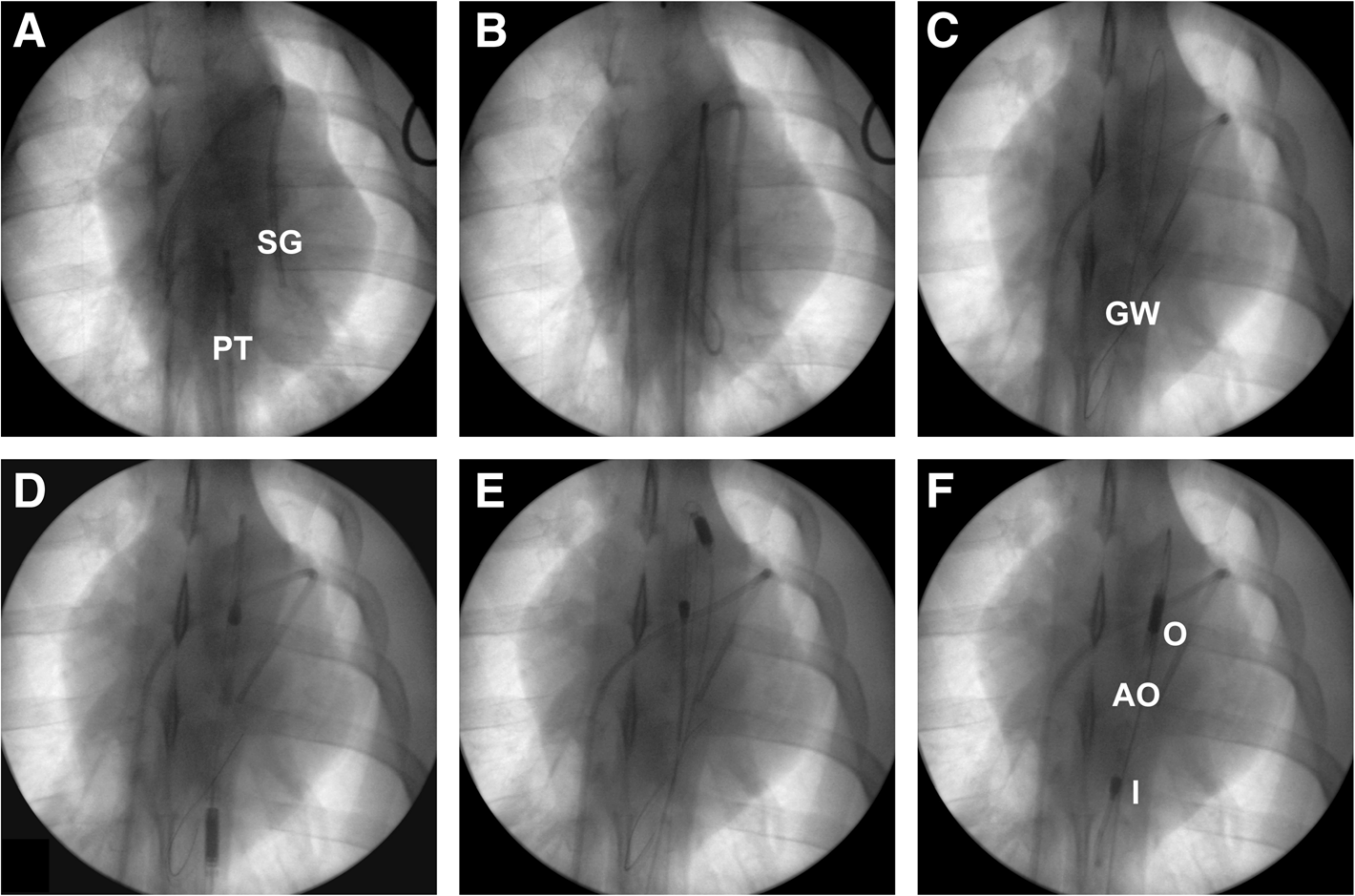
**Intravascular cardiopulmonary resuscitation device placement using fluoroscopy.** Intraventricular placement of the intravascular cardiopulmonary resuscitation (iCPR) device (modified Impella 2.5; Abiomed Europe GmbH, Aachen, Germany) before cardiac arrest and CPR as depicted by fluoroscopy (Ziehm Vision; Ziehm Imaging GmbH, Nuremberg, Germany). **(A)** A pigtail catheter is advanced into the descending (thoracic) aorta. SG, Swan-Ganz catheter; PT, Pigtail catheter. **(B)** The guidewire is advanced into the left ventricle via the pigtail catheter *in situ*. **(C)** Final position of the guidewire (GW) following removal of the pigtail catheter. **(D)** The iCPR device is advanced into the ascending aorta using the guidewire. **(E)** The iCPR device passes through the aortic arch. **(F)** Final position of the iCPR device with the tip situated in the left ventricle following the removal of the guidewire. I, Inlet; O, Outlet; AO, Approximate location of the aortic valve. Besides the materials mentioned and depicted above, a 13-French sheath introducer (Impella 2.5 introducer kit 13 F, 13 cm; Abiomed, Danvers, MA, USA) and an external microcontroller (Impella Controller; Abiomed) are required for placement and operation of the iCPR device.

For animals randomized to receive intravascular CPR (iCPR), a 13-French sheath introducer (Impella 2.5 introducer kit 13 F, 13 cm; Abiomed) was placed in the right femoral artery. Using this vascular access, a modified Impella 2.5 left ventricular assist device (Abiomed Europe GmbH, Aachen, Germany) equipped with a shortened angled cannula to meet the anatomical constraints of the animal was introduced into the left ventricle using fluoroscopy via a previously introduced pigtail catheter (Cordis 6 F PIG 145° 110 cm Super Torque Plus; Cordis, Miami Lakes, FL, USA) and a guidewire (Platinum Plus 0.018 in × 260 cm; Boston Scientific, Natick, MA, USA) (see Figure 2 for details).

For crossover experiments, a pigtail catheter was initially advanced into the left ventricle via the 13-French arterial sheath introducer to facilitate introduction of the Impella 2.5 after sCPR had failed.

All animals received two intravenous injections of 5,000 IU heparin; the first injection followed the completion of vascular access, and the second injection was administered shortly before the induction of VF to prevent blood clotting in catheters and introducer sheaths. To prevent wound infections, all animals received a single infusion of 1.5 g of cefuroxime during preparation.

### Experimental protocols

#### Chest compression vs. intravascular cardiopulmonary resuscitation

Cardiac arrest was induced with 1 to 2 mA of alternating current delivered to the endocardium of the right ventricle, resulting in VF. Simultaneously, mechanical ventilation was discontinued. Ten minutes following the onset of VF, CPR treatment was initiated, either using a piston-driven chest compressor (Thumper 1007; Michigan Instruments, Grand Rapids, MI, USA) at a rate of 100 compressions per minute as sCPR (n =10) or by activating the previously implanted Impella 2.5 at the maximum possible flow as iCPR (n =10). Chest compressions were adjusted to provide a compression depth of 25% of the chest diameter. In animals treated with sCPR, compressions were delivered unsynchronized with the simultaneously restarted mechanical ventilation, with equal compression–relaxation intervals (that is, a 50% duty cycle) and a compression depth of 25% of the chest diameter. In both groups, ventilation was adjusted to deliver a tidal volume of 15 ml/kg at a rate of 10/min. Concurrently, the FiO_2_ was increased to 1.0, and a bolus dose of 30 μg/kg epinephrine was injected into the right atrium via the Swan-Ganz catheter. An infusion of Ringer’s solution was started simultaneously via the femoral vein access at a rate of 100 ml/min to support left ventricular filling. A second dose of adrenaline (30 μg/kg) was administered 4 minutes and 30 seconds after the start of resuscitation efforts. After 6 minutes of sCPR or iCPR, defibrillation was attempted with up to two 200-J biphasic waveform shocks (M-Series CCT; Zoll Medical, Chelmsford, MA, USA) delivered between the left and right axillae. If an organized rhythm with a mean arterial pressure of greater than 60 mmHg persisted for 5 minutes, the animal was regarded as successfully resuscitated. If VF was not successfully reversed, 1 minute of sCPR or iCPR at the highest possible flow preceded the delivery of another sequence of up to two shocks.

After successful resuscitation, animals were monitored for 120 minutes. The FiO_2_ was kept at 1.0 for 30 minutes before prompt reduction to 0.3. In the iCPR group, the pump flow was adjusted to a rate of 1.5 L/min if ROSC had been achieved and was maintained for 60 minutes following ROSC. Thereafter, the flow was slowly reduced over 30 minutes, and the device was promptly removed. Hemostasis was assured by local manual pressure on the puncture site for 15 minutes. The incision above the left cephalic vein was closed with a suture.

To ensure adequate pain relief, all pigs received an intramuscular injection of 0.1 mg/kg buprenorphine 30 minutes before the end of the experiment. At the end of the observation period, the pentobarbital infusion was stopped, and animals were observed for signs of spontaneous breathing. Then, the ventilator was switched to assisted ventilation mode (continuous positive airway pressure assisted spontaneous breathing). When animals reached sufficient minute ventilation, a spontaneous breathing trial was attempted by disconnecting the respirator from the endotracheal tube. When the animal was able to maintain a peripheral capillary oxygen saturation above 96% and pCO_2_ below 45 mmHg at room air for 10 minutes, the cuff of the endotracheal tube was deflated and the animal was monitored for a minimum of another 5 minutes. If no depression in respiratory activity or blood gas values occurred, the endotracheal tube was then carefully removed. Following extubation, every animal was observed for at least 30 minutes to ensure adequate spontaneous breathing before being returned to its room.

#### Crossover experiments

In ten other animals, sCPR was stopped when the first two defibrillations at 200 J failed to terminate VF or resulted in asystole or pulseless electrical activity (PEA). Then the Impella 2.5 device was placed as described above (see Figure 2). iCPR was then initiated at the maximum flow capacity, and, simultaneously, another dose of adrenaline was injected. Two minutes thereafter, defibrillation at 200 J was attempted when appropriate. In other cases (for example, PEA), iCPR was maintained at the highest flow possible until either ROSC was achieved or 30 minutes of iCPR had passed without conversion into an organized rhythm. In the latter case, the animal was pronounced dead. In surviving animals, anesthesia was discontinued 120 minutes after ROSC to allow for gross neurological testing as described below, and the animals were killed thereafter.

#### Post-arrest care

All animals of the first protocol were visited at least in 6-hour intervals after being returned to their cages to observe clinical recovery. In cases where continuous presence of personnel was required, animals were observed permanently. If pigs were not able to rise, adequate positioning was ensured by turning the animals to minimize decubital ulceration of the skin. Furthermore, chow and water were offered by gentle spoon-feeding in cases where animals exhibited inadequate food intake. If an animal was not able to swallow, Ringer’s solution was administered intravenously at a rate of 10 ml/kg/hr. To allow for safe fluid administration, intravenous infusions were administered only when personnel were present. Pain relief was provided by an intramuscular injection of 0.1 mg/kg buprenorphine if tachypnea (>20/min) was observed or an animal seemed agitated. Animals that did not improve with this protocol and were not able to stand up and walk within 48 hours were killed by intravenous injection of a lethal dose of pentobarbital. All animals underwent systematic necropsy of the thoracic and abdominal cavities to identify injuries to the chest or thoracic or visceral organs.

### Measurements

Dynamic data, including heart rate, end-tidal CO_2_ (EtCO_2_), mean arterial pressure (MAP) and mean pulmonary artery pressure (MPAP), were continuously measured and digitally recorded (LabVIEW 2010, National Instruments, Austin, TX, USA; RedLab 1616HS-BNC, Meilhaus Electronic, Puchheim, Germany). Pump flow of the Impella 2.5 was determined using the corresponding controller (Automated Impella Controller, Software version 1.6; Abiomed) and recorded using the LabVIEW software. Cardiac output and blood temperature were continuously measured and recorded using a Vigilance monitor (Edwards Lifesciences).

Coronary perfusion pressure (CPP) was calculated by subtracting the mid-diastolic right atrial pressure from the mid-diastolic aortic pressure [21]. However, as the Impella does not generate pulsatile flow, no systolic or diastolic pressures are present during iCPR. Therefore, the non-pulsatile blood pressure was used for calculations during iCPR. As soon as pulsatile flow was present in animals treated with iCPR, diastolic pressures were used for CPP calculations.

The arterial oxygen and carbon dioxide tension (PaO_2_ and PaCO_2_, respectively), blood glucose levels and lactate levels were measured using a point-of-care blood gas analyzer (ABL 510; Radiometer, Copenhagen, Denmark). These measurements were obtained at baseline (BL) (that is, 5 minutes before cardiac arrest) and 10, 30, 60 and 120 minutes following ROSC.

At the same time points, serum samples were obtained to determine myoglobin (to quantify myocardial injury) and astroglial S100 protein levels (to quantify neuronal cell death) and promptly frozen to allow for measurements at a later time using commercially available enzyme-linked immunosorbent assay kits (S-100: YK150, BIOTREND Chemicals, Cologne, Germany; myoglobin: EIA3955, DRG International, Springfield, NJ, USA).

### Outcome testing

On each day post-arrest, animals were evaluated using a common clinical performance score (Overall Performance Categories (OPC)) as described in previous studies [18]. In brief, the test consists of five items representing the degree of impairment: OPC 1 is normal, with no obvious neurologic damage; OPC 2 represents moderate disability, with animals being conscious and aware, standing but unable to walk; OPC 3 is defined as severe disability, with animals being neither fully aware nor unconscious, but with reaction to pain and auditory stimuli, and not able to stand or walk; OPC 4 is coma; and OPC 5 is death or brain death. In animals that received buprenorphine as part of the post-arrest care protocol, an adequate interval (>4 hours) was allowed to elapse before neurological testing was performed. Neurological testing in the animals of the crossover experiments included an examination of spontaneous breathing, gait and pharyngeal and corneal reflexes.

### Statistical analysis

All data are expressed as the mean ± standard deviation unless stated otherwise. Normal distribution of the data was confirmed using the Kolmogorov-Smirnov test. For group comparisons of continuous variables, repeated-measures analysis of variance (ANOVA) was employed, followed by pairwise Student’s *t*-tests at given time points, adjusted for multiple comparisons by Bonferroni’s method in cases where significant differences were observed. Where appropriate, Fisher’s exact test was performed to compare categorical variables. In all cases, *P* ≤0.05 was considered to indicate statistical significance. IBM SPSS Statistics 21 software (Version 21.0.0.0; IBM, Armonk, NY, USA) was used for statistical calculations. GraphPad Prism software (Version 6.02; GraphPad Software, La Jolla, CA, USA) was used to create graphs.

## Results

No differences in hemodynamic or blood gas variables were observed between sCPR or iCPR animals at BL (Table 1).

**Table 1:** **Hemodynamics and blood gas data** ^**a**^

		**BL**	**PR 10**	**PR 30**	**PR 60**	**PR 120**
		**sCPR, n =10**	**sCPR, n =5**	**sCPR, n =5**	**sCPR, n =5**	**sCPR, n =5**
		**iCPR, n =10**	**iCPR, n =10**	**iCPR, n =10**	**iCPR, n =10**	**iCPR, n =10**
HR (bpm)	sCPR	109 ± 28	160 ± 23	169 ± 20	139 ± 21	115 ± 27
	iCPR	85 ± 25	162 ± 36	149 ± 32	112 ± 23	92 ± 12
MAP (mmHg)	sCPR	100 ± 15	94 ± 26	77 ± 29	84 ± 24	80 ± 18
	iCPR	96 ± 10	85 ± 22	88 ± 13	80 ± 18	89 ± 22
CO (L/min)	sCPR	5.8 ± 1.0	5.2 ± 1.9	5.5 ± 2.5	4.2 ± 1.9	3.1 ± 1.2
	iCPR	4.6 ± 1.1	6.1 ± 0.9	4.8 ± 0.7	3.2 ± 0.8	3.6 ± 1.2
MPAP (mmHg)	sCPR	19 ± 3	20 ± 3	19 ± 3	21 ± 3	20 ± 2
	iCPR	18 ± 3	19 ± 5	19 ± 2	20 ± 3	18 ± 4
PaO_2_ (mmHg)	sCPR	100 ± 10	520 ± 33	511 ± 64	104 ± 21	118 ± 26
	iCPR	107 ± 11	447 ± 90	433 ± 109	159 ± 76	136 ± 28
PaCO_2_ (mmHg)	sCPR	38 ± 3	41 ± 6	39 ± 3	39 ± 1	39 ± 3^b^
	iCPR	36 ± 3	42 ± 7	38 ± 6	35 ± 8	31 ± 4^b^
Lactate (mmol/L)	sCPR	2.7 ± 2.0	9.6 ± 3.3	9.2 ± 2.7	7.3 ± 2.8	4.6 ± 2.4
	iCPR	1.7 ± 0.8	8.1 ± 1.7	8.0 ± 2.2	7.5 ± 1.9	6.2 ± 3.2
Glucose (mmol/L)	sCPR	117 ± 19	309 ± 66	290 ± 83	241 ± 78	193 ± 57
	iCPR	129 ± 25	242 ± 76	225 ± 77	208 ± 61	178 ± 48
pH	sCPR	7.48 ± 0.03	7.27 ± 0.09	7.30 ± 0.05	7.35 ± 0.06	7.40 ± 0.05
	iCPR	7.46 ± 0.03	7.27 ± 0.09	7.30 ± 0.08	7.36 ± 0.07	7.44 ± 0.08

^a^Hemodynamic and blood gas data in 20 pigs treated either with standard cardiopulmonary resuscitation (sCPR; n =10) or an intravascular CPR device (iCPR; n =10) at baseline (BL) or 10 (PR10), 30 (PR30), 60 (PR60) or 120 (PR120) minutes following return of spontaneous circulation. All animals received a bolus dose of 30 μg/kg epinephrine intravenously twice during CPR. HR, Heart rate; MAP, Mean arterial pressure; CO, Cardiac output; MPAP, Mean pulmonary artery pressure; PaO_2_, Arterial oxygen tension; PaCO_2_, Arterial carbon dioxide tension. Mean ± standard deviation. ^b^ *P* ≤0.01, iCPR vs. sCPR.

**Figure 3:**
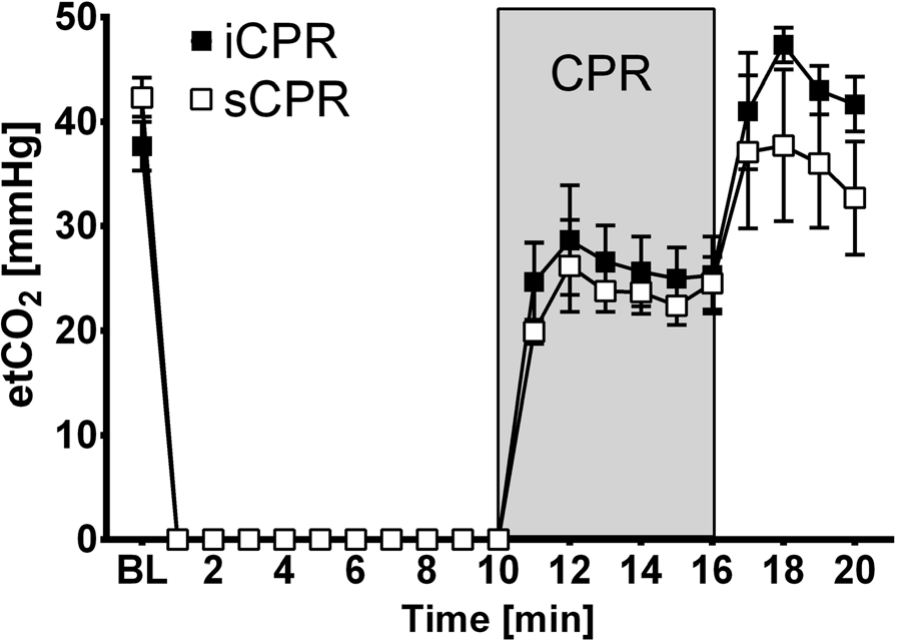
**End-tidal carbon dioxide pressure in animals treated with intravascular cardiopulmonary resuscitation vs. standard cardiopulmonary resuscitation.** End-tidal carbon dioxide pressure (etCO_2_) in animals treated with standard chest compression (sCPR) (n =10) or the intravascular cardiopulmonary resuscitation (iCPR) device (n =10). Data are presented as the mean ± standard error of the mean. BL, Baseline (that is, 5 minutes prior to cardiac arrest).

**Figure 4:**
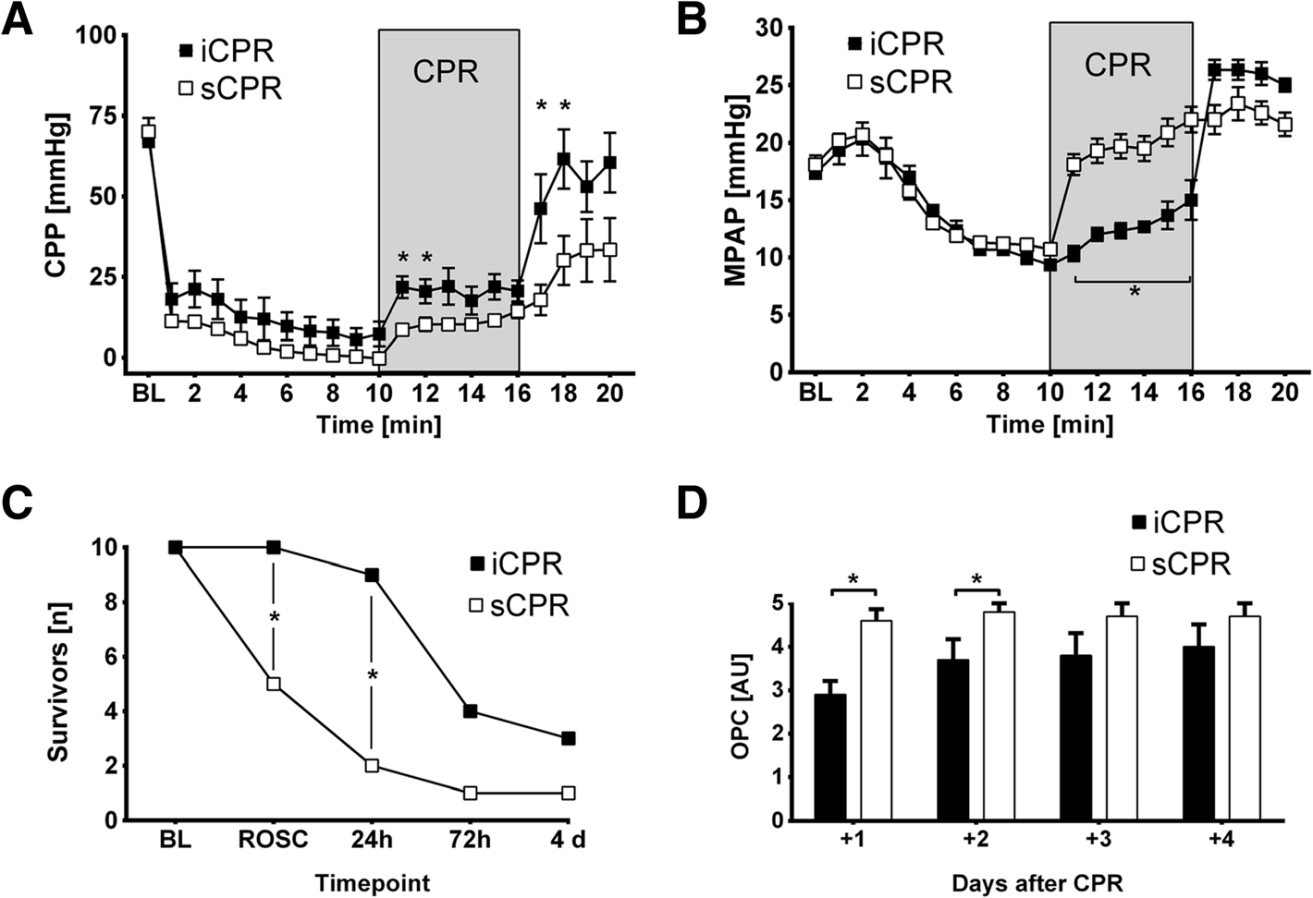
**Standard cardiopulmonary resuscitation vs. intravascular cardiopulmonary resuscitation.** Comparison of standard cardiopulmonary resuscitation (sCPR) (n =10) or intravascular cardiopulmonary resuscitation (iCPR) (n =10). Data are presented as the mean ± standard error of the mean. *P ≤0.05, comparing sCPR vs. iCPR. BL, Baseline (that is, 5 minutes prior to cardiac arrest). **(A)** Calculated coronary perfusion pressure (CPP). **(B)** Mean pulmonary artery pressure (MPAP). **(C)** Survival data. Animals with severe neurocognitive outcomes were killed 48 hours post-CPR (n =5) or when they were not able to stand or walk (n =1). ROSC, Return of spontaneous circulation. **(D)** Overall Performance Categories (OPC). OPC 1, Normal; no obvious neurologic damage; OPC 2, Moderate disability; animals being conscious and aware, standing but unable to walk; OPC 3, Severe disability; animals being neither fully aware nor unconscious, but with reaction to pain and auditory stimuli, not able to stand or walk; OPC 4, Coma; OPC 5, Death or brain death.

Although there was a trend toward higher EtCO_2_ values in iCPR-treated animals, this trend did not reach statistical significance (Figure 3). In contrast, we observed a rise in CPP when iCPR was initiated, which resulted in significantly higher values than those in sCPR animals over the first 2 minutes (*P* <0.01 for 11 minutes after VF; *P* <0.05 for 12 minutes after VF) (Figure 4A). In addition, iCPR-treated animals exhibited significantly lower MPAP, which dramatically increased during chest compression in sCPR animals (*P* <0.01 for 11 to 16 minutes after VF) (Figure 4B). This consistent increase in CPP, in concert with the effectively lower MPAP, translated into a high rate of ROSC, as all animals in the iCPR group were successfully resuscitated, compared with only 50% in the sCPR group (*P* =0.03) (Figure 4C).

Although dosing of epinephrine did not differ significantly between sCPR and iCPR treatment (2.4 ± 1.0 mg vs. 2.8 ± 0.8 mg; *P* =0.38), we observed a significantly higher number of shocks in sCPR-treated animals, even when we excluded animals that did not achieve ROSC (3.4 ± 1.7 shocks vs. 1.6 ± 1.1 shocks; *P* <0.01). Although sCPR treatment tended to require more time before ROSC was achieved, this notion did not reach statistical significance (16.9 ± 1.2 minutes vs. 19.6 ± 5.0 minutes; *P* =0.16).

Animals in both groups showed typical signs of global ischemia–reperfusion injury, with pronounced tachycardia and subsequent reductions of BL cardiac output values following successful CPR (see Tables 1 and 2). In addition to a significant difference in PaCO_2_ between iCPR- and sCPR-treated animals 2 hours post-ROSC (*P* <0.01), which was attributable to mild hyperventilation in iCPR animals at the end of the observation period, no differences in post-arrest hemodynamics or blood gas variables were observed (Table 1).

**Table 2:** **Hemodynamics and blood gas data in crossover experiments** ^**a**^

	**BL, n =10**	**PR 10, n =6**	**PR 30, n =6**	**PR 60, n =6**	**PR 120, n =6**
HR (bpm)	101 ± 15	158 ± 44	148 ± 40	124 ± 39	134 ± 31
MAP (mmHg)	113 ± 9	86 ± 21	65 ± 14	69 ± 18	75 ± 21
CO (L/min)	5.4 ± 1.2	5.6 ± 1.1	4.2 ± 2.5	2.3 ± 0.6	2.8 ± 1.2
MPAP (mmHg)	20 ± 5	21 ± 6	17 ± 4	19 ± 5	21 ± 4
PaO_2_ (mmHg)	104.3 ± 9.5	492.8 ± 54.3	487.0 ± 39.7	109.8 ± 17.6	112.1 ± 12.0
PaCO_2_ (mmHg)	41.5 ± 3.6	45.5 ± 7.2	38.0 ± 3.9	38.3 ± 5.2	34.3 ± 3.1
Lactate (mmol/L)	1.2 ± 1.1	7.8 ± 1.4	7.8 ± 1.7	7.2 ± 2.0	5.7 ± 2.8
Glucose (mmol/L)	105 ± 6	209 ± 85	201 ± 59	197 ± 47	186 ± 44
pH	7.46 ± 0.04	7.22 ± 0.09	7.31 ± 0.05	7.37 ± 0.07	7.40 ± 0.05

^a^Hemodynamic and blood gas data in ten pigs treated with intravascular cardiopulmonary resuscitation after failure of standard cardiopulmonary resuscitation at baseline (BL) or at 10 (PR 10), 30 (PR 30), 60 (PR 60) or 120 (PR 120) minutes following return of spontaneous circulation. All animals received a bolus dose of 30 μg/kg epinephrine intravenously twice during cardiopulmonary resuscitation. HR, Heart rate; MAP, Mean arterial pressure; CO, Cardiac output; MPAP, Mean pulmonary artery pressure; PaO_2_, Arterial oxygen tension; PaCO_2_, Arterial carbon dioxide tension. Data are presented as the mean ± standard deviation.

iCPR yielded only approximately 50% of the maximum pump flow capacity of 2.5 L/min (1.36 ± 0.02 L/min; minimum, 0.87 L/min; maximum, 2.28 L/min). Nevertheless, 24 hours after resuscitation, the mortality rate in the sCPR group exceeded 50%, in contrast to the mortality rate of only 10% in the iCPR group (Figure 4C). Clinically, all animals in the sCPR group exhibited severe neurological dysfunction, which was significantly less in the iCPR-treated animals (*P* <0.01, sCPR vs. iCPR at both day 1 and day 2) (Figure 4D). However, owing to significant neurological impairment in both groups, several animals had to be killed during the observation period, resulting in diminished differences in both outcomes and mortality on days 3 and 4 after resuscitation (see Figures 4C and D for further details).

**Figure 5:**
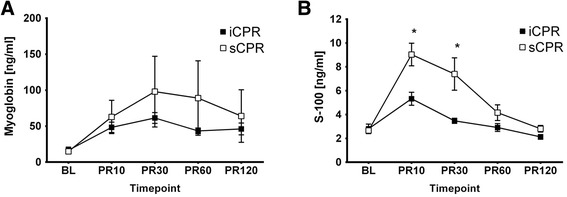
**Biomarkers.** Serum values of animals treated with standard cardiopulmonary resuscitation (sCPR) (n =10) or intravascular cardiopulmonary resuscitation (iCPR) (n =10) before CPR at baseline (BL) or at 10 (PR10), 30 (PR30), 60 (PR60), or 120 (PR120) minutes following return of spontaneous circulation. Data are presented as the mean ± standard error of the mean. *Significant difference between iCPR and sCPR. **(A)** Myoglobin serum levels were used as a correlate of myocardial ischemia. **(B)** Serum S100 levels were employed as an indicator of the degree of cerebral ischemia–reperfusion injury.

While serum myoglobin levels were comparable between iCPR and sCPR animals up to 2 hours post-CPR, animals in the iCPR group had significantly lower serum S100 levels compared with animals that received sCPR 10 and 30 minutes following ROSC (*P* <0.01, sCPR vs iCPR at 10 and 30 minutes after ROSC) (Figure 5B).

**Figure 6:**
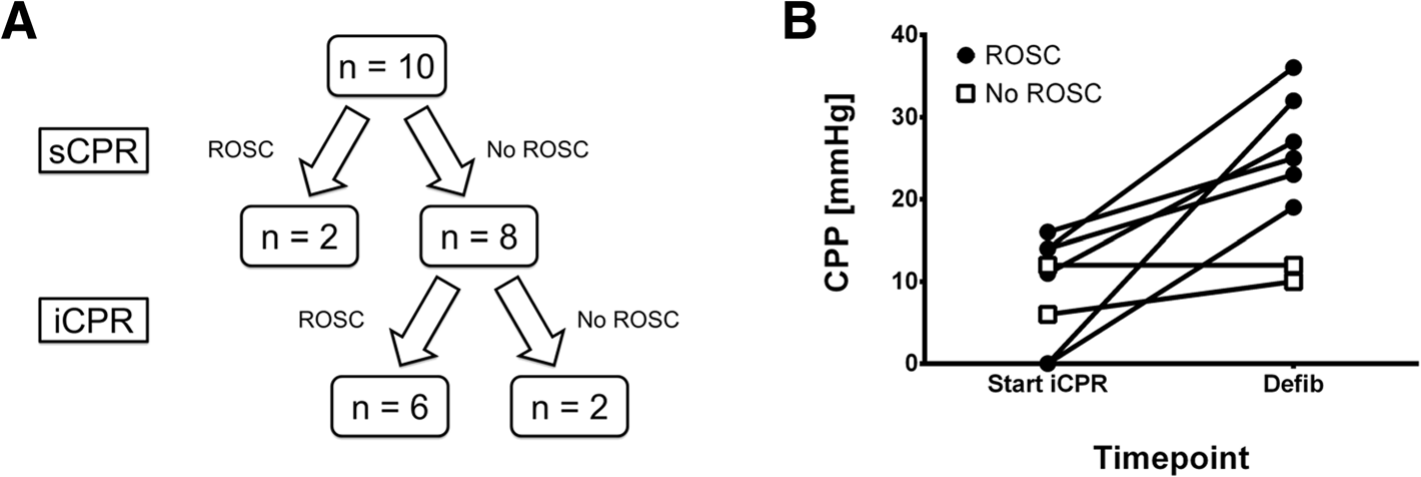
**Crossover experiments.** Crossover experiments in ten pigs using the intravascular cardiopulmonary resuscitation device (iCPR) following failed standard CPR (sCPR). Data are presented as the mean ± standard error of the mean. **(A)** Survival data. **(B)** Calculated coronary perfusion pressure (CPP) in eight pigs that either developed no return of spontaneous circulation (no ROSC, n =2) or developed spontaneous circulation (ROSC, n =6) following treatment with iCPR. Values estimated 30 seconds after implantation of the iCPR device (Start iCPR) or following at least 2 minutes of iCPR treatment, 30 seconds prior to defibrillation (Defib).

In crossover experiments, eight of ten animals did not achieve ROSC after sCPR and were further treated with iCPR. Notably, of these eight animals, six were successfully resuscitated (Figure 6A) and exhibited a twofold increase in CPP values 30 seconds prior to defibrillation, as shown in Figure 6B. Animals that did not achieve ROSC with sCPR but did with iCPR (n =6) required, on average, 3.3 ± 0.9 mg of epinephrine and 7.3 ± 3.9 shocks before ROSC was achieved.

Implanting the iCPR device following failed sCPR was performed within 61 ± 72 seconds, but led to prolonged periods until ROSC was achieved in crossover experiments (22.8 ± 5.1 minutes for sCPR + iCPR vs. 16.9 ± 1.4 minutes for iCPR only; *P* ≤0.01). However, all animals resuscitated with iCPR following failed sCPR exhibited brainstem functions, such as spontaneous breathing, gaiting and pharyngeal and corneal reflexes, after anesthesia was ceased.

Necropsy revealed no macroscopically visible lesions in the heart or major vessels, but it did show multiple rib fractures in all sCPR-treated animals. No hemato- or pneumothoraxes were found in any of the animals.

## Discussion

Our study demonstrates that, during experimental cardiac arrest, the use of iCPR provides significantly improved CPP with concurrent significant reductions in MPAP values, translating into good resuscitation outcomes. The iCPR approach doubled survival and resulted in a favorable overall outcome compared with sCPR. We further demonstrated that iCPR following 6 minutes of futile sCPR was still effective in 75% of the animals that would most likely have died otherwise.

### Benefits of mechanical circulatory support during cardiopulmonary resuscitation

Resuscitation guidelines were first published in the 1970s and were based on the fundamental findings of the importance of chest compression and artificial ventilation [22]. Major changes primarily highlighting the importance of early defibrillation and post-resuscitation care followed [23]. However, ultimate outcomes have not been consistently improved, which may be partly explained by the way forward blood flow is generated during sCPR. Chest compressions are at best able to deliver 20% to 30% of the stroke volume of a healthy human [24] and roughly 30% of BL cardiac output in animals [6]. In addition, rescuers deliver chest compressions inconsistently [7,25] or become fatigued, thereby further decreasing resuscitation success [26]. However, the use of mechanical devices that provide sustained high-quality chest compressions have yielded mostly disappointing results [9,10]. From a mechanistic point of view, forward blood flow generated by chest compressions may, in general, offer a suboptimal approach to overcoming ischemic insults because only depth and rate can be controlled. Furthermore, coronary arteries are perfused only during decompression (as they are perfused only during diastole in the beating heart). As a consequence, adequate coronary flow is provided during only 50% of the CPR cycle. These hemodynamic properties are significantly different in iCPR, during which a constant non-pulsatile flow is created. Thus, it is presumable that coronary perfusion with iCPR is superior to sCPR, which is at least partly underscored by the faster increase of CPP values following initiation of CPR compared with sCPR-treated animals. Furthermore, coronary perfusion is dependent on not only the arteriovenous gradient, as suggested by CPP calculations. It has been well described that unloading the left ventricle with a left ventricular assist device leads to increased coronary perfusion by lowering wall stress in the coronary arteries [27], as well as to reduced myocardial injury following reperfusion [28].

From this perspective, other approaches in which several factors influencing the magnitude of reperfusion injury can be modified are more promising in improving resuscitation success. For example, ECMO offers the possibility to effectively control not only blood flow but also temperature and the content of the reperfusate. In experimental settings, using ECMO in CPR is highly effective and yields good resuscitation outcomes, even after very prolonged cardiac arrest [29]. In specialized centers, early ECMO therapy has been shown to improve ROSC rates in in-hospital cardiac arrest patients who do not respond to sCPR [14], and further benefits due to technical refinements and miniaturization may be anticipated [30]. However, in out-of-hospital patients, such promising results were not obtained [31,32]. This result can be explained in part by the highly invasive nature of the procedure, which requires time-consuming cannulation of two large vessels, increasing the risk of cannula misplacement, vascular dissection and limb ischemia. Furthermore, patients treated with ECMO are prone to sepsis, bleeding and renal failure [33].

The Impella system may offer significant advantages over such approaches, as only single-vessel access is required. Furthermore, no external circuit is needed, and blood flow from the left ventricle into the ascending aorta is more likely to perfuse the heart and brain than is the case with ECMO, with the flow directed retrogradely into the abdominal aorta [34]. Furthermore, we observed significantly lower pressures in the pulmonary artery in iCPR, which was most likely due to the intraventricular uptake of blood by the iCPR device. This was an unanticipated effect, which may sufficiently explain improved ROSC rates in iCPR animals, as it has been reported that elevated right ventricular pressures significantly impair coronary blood flow [35]. However, this ventricular unloading did not result in higher blood flow values, as documented by EtCO_2_ during CPR, or greater cardiac output following ROSC.

Although the use of the Impella system has been extensively described in several cases of advanced cardiac failure [36], there are only a few reported studies of its use in the context of cardiac arrest, predominantly in post-cardiac arrest patients presenting with low cardiac output syndrome or recurring episodes of VF [37]. In three animal studies, Tuseth and colleagues showed that cerebral and myocardial perfusion could be significantly improved compared with open-chest cardiac compressions for up to 45 minutes [16,38,39]. However, they did not report whether this therapy had an influence on survival or neurological outcome in a closed-chest model of cardiac arrest and CPR. Our results clearly demonstrate a dramatic improvement in resuscitation efficacy using the iCPR approach, even after conventional CPR failed and ischemia time exceeded 20 minutes. These clinical results were reflected by lower serum markers for cerebral injury in the early post-resuscitation period and a trend toward less myocardial injury, as detected with serum myoglobin levels. The lack of a significant difference in serum myoglobin levels shows that the degree of myocardial injury appears to be dominated by ischemia, not mechanical trauma in our setting.

Although our results have the potential to introduce a new paradigm in resuscitation science, they should not be considered to imply a less important role for conventional chest compressions in CPR. Chest compressions remain a simple and readily available technique by which to provide circulatory support that can buy time until the appropriate therapy, such as defibrillation or percutaneous coronary intervention, can be established. However, the iCPR intervention proposed here may be more suitable than sCPR in specific populations or circumstances, such as prolonged CPR in hypothermic or intoxicated patients, cardiac arrest in the catheter laboratory, or following recent sternotomy.

### Translation into clinical practice

Several issues need to be addressed before direct translation of the iCPR approach to the clinical setting can occur. The flow delivered by the device was only 50% of the maximum capacity of 2.5 L, which may be explained by the smaller size of the left ventricular cavity in swine hearts compared with human hearts. Furthermore, although we generously administered intravenous fluid during CPR, left ventricular filling may have been suboptimal, as iCPR treatment does not feature variations in intrathoracic pressure and may therefore result in lower venous return than occurs during sCPR. In this context, it is noteworthy that during CPR, pulmonary artery pressure and pulmonary vascular resistance are known to be almost threefold higher than normal, thus limiting right-to-left-sided blood flow in this setting [40,41]. Strategies to promote blood flow from the right to the left side of the heart by administering inhaled pulmonary vasodilators, such as nitric oxide or prostacyclin, during CPR may therefore be a promising strategy for increasing left ventricular filling and thus stroke volume [42]. However, the lower MPAP values in this investigation suggest that using the iCPR approach *per se* may facilitate right-to-left-sided blood flow. Additionally, augmentation of systemic vascular resistance with the aim of increasing the perfusion of vital organs, such as the brain, heart and kidneys, may be further enhanced if the iCPR device is equipped with a balloon partially occluding the descending aorta [43].

Another important consideration is vascular access and placement of the device under clinical conditions. Identifying and successfully puncturing the femoral artery in cardiac arrest patients, most of whom have underlying calcifications of large vessels, are more complicated than under the controlled circumstances of our laboratory investigation. This is also true for the correct placement of the device in the left ventricle, which was performed under fluoroscopic guidance. All these obstacles may limit the use of the iCPR strategy to certain clinical settings. However, the value of this approach may be even higher in the preclinical setting, with the aim of promoting vital organ blood flow as early as possible, as this has been identified as one of the key factors for enhancing cardiac arrest outcomes [44,45]. Gaining arterial access has been found to be safe and feasible in the prehospital setting and may be enhanced by using ultrasound [46,47]. Emergency sonography of the heart may also guide placement of the Impella device [48].

Translation of the iCPR approach to the preclinical arena may therefore be more feasible than one would assume based on today’s Impella procedures and protocols.

### Limitations

We recognize several limitations when interpreting our results. First, the results were obtained from healthy animals, which precludes the direct translation of results to humans, many of whom present with underlying diseases.

Second, we did not directly measure cerebral or coronary blood flow during CPR, which has been extensively reported by Tuseth and colleagues in an open-chest cardiac arrest model [16,38,39]. However, because we decided to focus on resuscitation success and clinical outcomes, we were obliged to avoid invasive measurements that may have compromised survival itself.

Third, others have reported higher CPP values in similar experimental settings when using advanced CPR-augmenting techniques such as active decompression CPR or an impedance threshold device. The quality of CPR in the sCPR group may therefore have potential for improvement. However, these devices are not widely used in clinical practice, as they both increase the workload during CPR and have yet to show whether they contribute to improved survival. We therefore opted not to use these in the current investigation to reflect clinical reality in our model.

Furthermore, it remains unclear whether the cardiac support provided by iCPR following ROSC contributed to the favorable neurological outcomes in these animals. This may very well be the case, although neither EtCO_2_ nor cardiac output showed a significant difference between surviving sCPR and iCPR animals in the early or late post-CPR period. Cardiac support with the Impella device may have contributed to the better outcome, as it may have prevented rearrest due to better coronary perfusion in the early post-arrest period in iCPR-treated animals. As patients frequently present with sustained arrhythmias following ROSC, this may be a possible advantage over chest compression CPR, where cardiac support stops as soon as chest compressions are suspended. However, others have found that cardiac support in heart failure with an intra-aortic balloon pump [49] or an implantable device in post-myocardial infarction cardiogenic shock [50] did not improve outcomes in clinical trials.

It is of note that survival at 72 hours and 4 days was not improved in this investigation. However, animals did not receive advanced neuroprotective treatments such as mild therapeutic hypothermia, temperature control, or neuroprotective drugs such as isoflurane or noble gases. Combining the improved primary survival with the Impella device with such treatments to improve long-term functional outcome therefore warrants further studies and is within the scope of future investigations by our group.

Last, collection of survival and neurocognitive outcome data has been constrained by early euthanasia in cases where animals were not able to stand within 36 hours after CPR. Other concepts of care may have helped to translate promising results from the early post-arrest period into improved 4-day survival and outcome.

## Conclusions

Our results demonstrate that, in a clinically relevant, large animal model of cardiac arrest and CPR, iCPR treatment was superior to sCPR with respect to both the ability to achieve ROSC and the ability to achieve a better functional outcome in the early post-arrest period. Furthermore, iCPR restored spontaneous circulation in the majority of cases in which ROSC could not be achieved by sCPR. These results suggest a possible role for iCPR treatment in cardiac arrest that has, to our knowledge, not been previously proposed. Further studies are warranted to determine the feasibility of using this strategy in different clinical settings to evaluate its potential in humans.

## Key messages

- iCPR is superior to sCPR in regard to ROSC and clinical outcome in a porcine model of cardiac arrest and CPR.
- Optimizing CPP and MPAP with iCPR improves ROSC rates, even when the device is placed following unsuccessful sCPR.
- Verification of correct device placement currently requires fluoroscopy, restricting the deployment of iCPR to a limited number of clinical settings.
- Modification of the device and procedure is required for its use beyond the catheter laboratory.

## Abbreviations

ANOVA: Analysis of variance
BL: Baseline
CA: Cardiac arrest
CO: Cardiac output
CPP: Coronary perfusion pressure
CPR: Cardiopulmonary resuscitation
Defib: Defibrillation
ECMO: Extracorporeal membrane oxygenation
EtCO_2_: End-tidal carbon dioxide
FiO_2_: Inspired oxygen fraction
HR: Heart rate
iCPR: Intravascular cardiopulmonary resuscitation
IV: Intravenous
MAP: Mean arterial pressure
MPAP: Mean pulmonary artery pressure
OPC: Overall Performance Categories
PaCO_2_: Arterial carbon dioxide tension
PaO_2_: Arterial oxygen tension
pCO_2_: Carbon dioxide tension
PEA: Pulseless electrical activity
ROSC: Return of spontaneous circulation
sCPR: Standard cardiopulmonary resuscitation
VF: Ventricular fibrillation
